# Exposure to volatile organic compounds and airway inflammation

**DOI:** 10.1186/s12940-018-0410-1

**Published:** 2018-08-07

**Authors:** Jae-Woo Kwon, Hee-Won Park, Woo Jin Kim, Man-Goo Kim, Seung-Joon Lee

**Affiliations:** 1 Department of Allergy and Clinical Immunology, Kangwon National University Hospital, Chuncheon, South Korea; 2 Department of Internal Medicine, Kangwon National University College of Medicine, Baengnyeong-ro 256, 200-722 Chuncheon-Si, Gangwon-Do, Chuncheon, South Korea; 3 Environmental Health Center, Kangwon National University Hospital, Chuncheon, South Korea; 4 Department of Rehabilitation Medicine, Kangwon National University College of Medicine, Chuncheon, South Korea; 5 Department of Environmental Science, Kangwon National University, Chuncheon, South Korea

**Keywords:** Volatile organic compounds, Sick building syndrome, Oxidative stress, Inflammation, Spirometry

## Abstract

**Background:**

Exposure to low levels of volatile organic compounds (VOCs) in ordinary life is suspected to be related to oxidative stress and decreased lung function. This study evaluated whether exposure to ambient VOCs in indoor air affects airway inflammation.

**Methods:**

Thirty-four subjects from the hospital that had moved to a new building were enrolled. Symptoms of sick building syndrome, pulmonary function tests, and fractional exhaled nitric oxide (FeNO) were evaluated, and random urine samples were collected 1 week before and after the move. Urine samples were analyzed for VOC metabolites, oxidative stress biomarkers, and urinary leukotriene E4 (uLTE4) levels.

**Results:**

The level of indoor VOCs in the new building was higher than that in the old building. Symptoms of eye dryness and eye irritation, as well as the level of a xylene metabolite (o-methylhippuric acid) increased after moving into the new building (*p* = 0.012, *p* = 0.008, and *p* < 0.0001, respectively). For the inflammatory markers, FeNO decreased (p = 0.012 and *p* = 0.04, respectively) and the uLTE4 level increased (*p* = 0.005) after the move.

**Conclusion:**

Exposure to a higher level of VOCs in everyday life could affect airway inflammation.

**Electronic supplementary material:**

The online version of this article (10.1186/s12940-018-0410-1) contains supplementary material, which is available to authorized users.

## Background

Indoor air pollution is attracting attention as people spend more time indoors. Volatile organic compounds (VOCs) are important indoor air pollutants produced by evaporation at room temperature from diverse sources, such as building materials, paints, cleaning agents, furnishings, adhesives, combustion materials, floor, and wall coverings [1]. Greater use of VOC-containing products, more effective insulation, and less external ventilation of modern buildings has contributed to increased VOC exposure. VOCs are known to be associated with respiratory symptoms in industrial or occupational environments [2], but the effects of exposure to low concentrations on the airway in the general indoor environments of daily life are not clear.

Sick building syndrome (SBS) is associated with various health problems that patients may report with an unclear cause, but with a possible relationship to the indoor environment [3]. However, the causal relationship between SBS and the indoor environment was debated until Lu et al. reported the relationship between exposure to VOCs, SBS symptoms, and oxidative stress among office workers [4]. Yoon et al. showed that urinary levels of VOC metabolites are related with oxidative stress and decreased lung function [5]. However, the mechanism of how VOCs and oxidative stress are related to decreased lung function is unclear. No studies have been performed on airway inflammation induced by exposure to VOCs during ordinary life, and longitudinal studies about SBS are rare, although there are a large number of cross-sectional studies [6].

We hypothesized that exposure to VOCs promotes airway inflammation. We evaluated SBS symptoms, urinary levels of VOC metabolites and oxidative stress markers, lung function, and inflammatory markers including fractional exhaled nitric oxide (FeNO) and urine leukotriene E4 (uLTE4) of subjects before and after moving into a new building with a relatively high level of ambient VOCs.

## Methods

### Study subjects

Study populations were enrolled from the inpatients, caregivers, and workers at a 60-bed rehabilitation hospital that moved to a new building. This is a hospital for patients in daily physical rehabilitation programs and without acute or chronic severe illness. Inclusion criteria were staying in the hospital > 8 h/day for more than 1 week. Exclusion criteria were symptoms of upper respiratory infection within 1 week and any illness that could affect airway inflammation and lung function. The move-in building was newly built and located near the old building within a 900 m straight line, and thus the new building shared almost the same outdoor environment. The old building was located 70 m from a two-lane low-traffic road, whereas the new building was located 200 m from a four-lane low-traffic road.

**Fig. 1:**
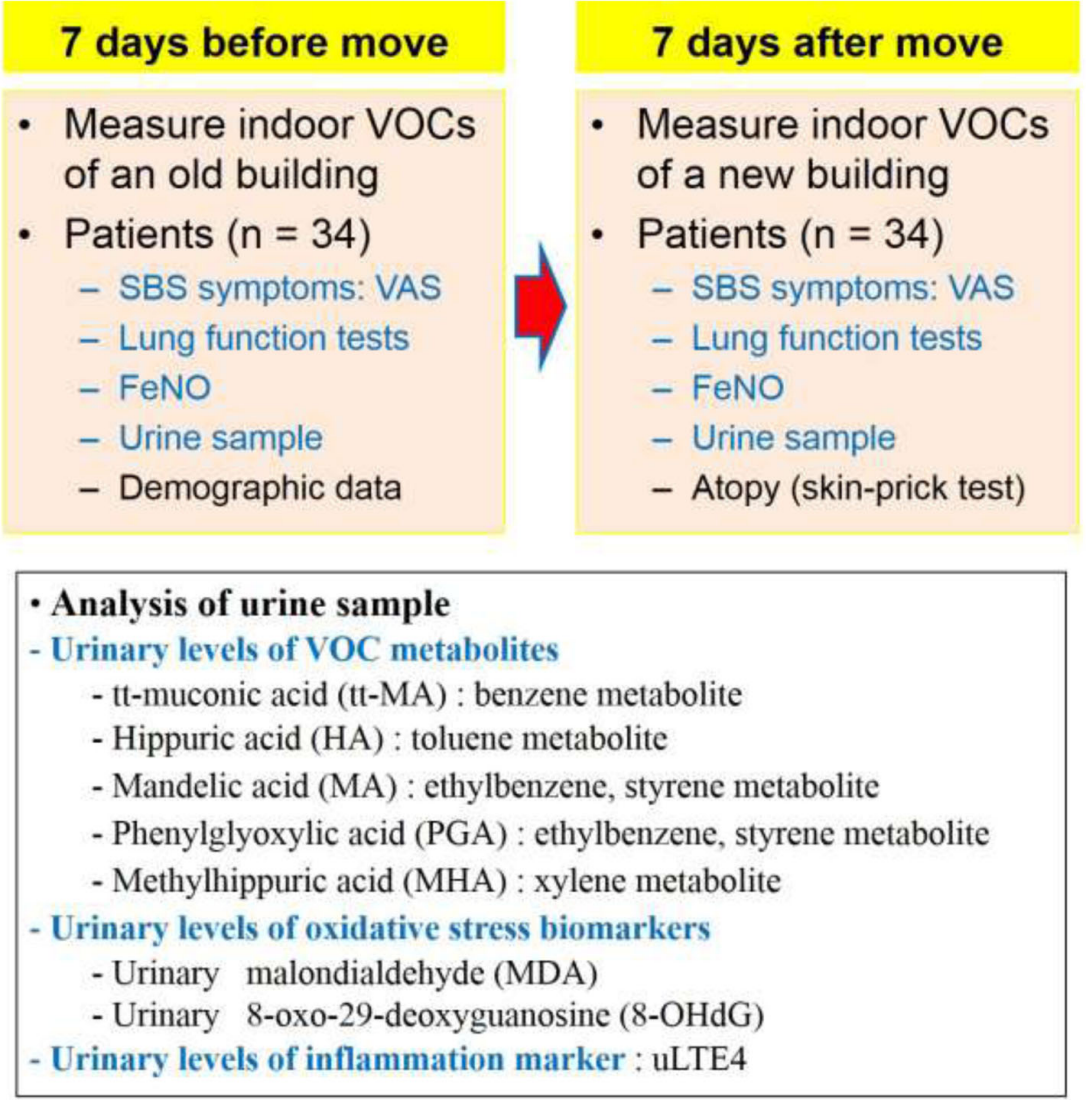
Study design

Demographic characteristics, smoking status, history of allergic diseases, including asthma, allergic rhinitis, atopic dermatitis, food allergy, and drug allergy, and a family history of allergic diseases, were collected. SBS symptoms, lung function tests, and FeNO were evaluated, and spot urine samples were collected 7 days before and after the move (Fig. 1). SBS symptoms included a runny nose, stuffy nose, sneezing, headache, eye dryness, eye irritation, tiredness, skin dryness, chest discomfort, breathing difficulties, sputum, cough, hypersensitivity to pollutions or irritants, wheezing, and any sleeping difficulties [4], and symptoms were evaluated via a visual analogue scale written on a paper questionnaire. FeNO, usually regarded as marker of T-helper cell type 2 (Th2) airway inflammation, was measured using a Niox Mino (Aerocrine, Solna, Sweden) according to American Thoracic Society/European Respiratory Society recommendations by one experienced operator [7]. Spirometry was performed using an Easy One kit (NDD; Zurich, Switzerland), as recommended by the American Thoracic Society/European Respiratory Society [8]. Spot urine samples were collected from all subjects in the afternoon of each day. Skin-prick tests were performed for 15 common inhalant allergens 7 days after the move using a test kit that included a battery of 15 common inhalant allergens (Allergopharma Co., Reinbek, Germany), a positive control (2 mg/mL histamine) and negative diluent controls (Allergopharma). A mean wheal diameter ≥ 3 mm was regarded as a positive reaction. Meteorological and air pollution data for 6 days before and at each evaluation date were collected for the outdoor environment. Meteorological data, including daily highest and mean temperature and mean relative humidity were retrieved from the Korean Meteorological Administration database. Data about air pollution retrieved from routine monitoring of gaseous air pollutants by the Gangwon Institute of Health and the Environment included the daily average levels of sulfur dioxide, nitrogen dioxide, ozone, carbon monoxide, and airborne particulate matter ≤10 μm in diameter. All of the study subjects signed a written informed consent form. All study procedures and the participants were in accordance with the Declaration of Helsinki (1964) and its later amendments.

### Ambient levels of VOC

We measured indoor levels of VOCs, formaldehyde, and acetaldehyde in the old building and the new building just before moving. The total levels of VOCs and those of five individual VOCs, including benzene, toluene, ethylbenzene, styrene, and o-, m-, and p-xylenes were measured. Ambient levels of indoor VOCs and aldehydes were measured at two locations in the old and new buildings, respectively. Duplicate samples were analyzed for each location, and the average values were used as typical concentrations of pollutants in the old and new buildings. After calibration, the sampling equipment was placed > 1 m from the wall and 1.5 m above the floor in the admission room. The room was ventilated for 30 min by opening the windows and doors to the room and then sealed for 1 h by closing the windows and doors of the room but opening the doors for storing furniture in the room. Air sampling was done by collecting VOCs and aldehydes simultaneously with four sampling lines. Duplicate VOC samples were collected on Tenax-TA sorbent tubes at 100 ml/min for 60 min and analyzed by gas chromatography/mass spectroscopy according to the ISO 16000–6 method. Duplicate aldehyde samples were collected on DNPH cartridges with 500 ml/min for 60 min and analyzed by high performance liquid chromatography according to the ISO 16000–3 method.

### Analysis of urine sample

#### Urinary levels of VOC metabolites and oxidative stress biomarkers

Hippuric acid (HA), tt-muconic acid (tt-MA), and o-, m-, and p-methylhippuric acids (o-, m- and p-MHAs) were measured as major metabolites of toluene, benzene, and o-, m-, and p-xylenes, respectively [9]. Mandelic acid (MA) and phenylglyoxylic acid (PGA), which are two major metabolites of styrene and are metabolites of ethylbenzene, were measured in urine. In addition, urinary malondialdehyde and 8-oxo-29-deoxyguanosine levels were determined to evaluate oxidative stress [5]. Details of the measurement methods are provided in the online supplement. The urinary levels of the VOC metabolites and oxidative stress markers were adjusted for urine creatinine.

#### Urinary level of LTE4

Urinary LTE4 concentrations were measured using a LTE4 enzyme-linked immunosorbent assay kit (No. 520411; Cayman Chemical, Ann Arbor, MI, USA,) as described previously [10, 11].

### Statistical analysis

Baseline characteristics of the study population and the biochemical analysis of the urine sample are presented as medians (range) for continuous variables and as relative frequencies for categorical variables. The changes in variables before and after the move were analyzed with Wilcoxon matched pairs rank test or Spearman’s rank correlation coefficient analysis, as the variables did not follow a normal distribution. The statistical analysis was performed using the SPSS version 22.0 for Windows software package (SPSS Inc., Chicago, IL, USA). A *p*-value < 0.05 was considered significant.

## Results

A total of 34 people were enrolled before the move and were followed for 7 days after the move. The demographic characteristics of the study population are shown in Table 1. There were 11 workers, 14 caregivers, and 9 patients who had been admitted to the hospital for the daily rehabilitation program but had no acute or severe chronic illness. The levels of ambient VOCs were higher in the new building than those in the old building (Table 2). No large differences in daily mean temperature or air pollution were observed before or after the move (Additional file 1: Table S1).

**Table 1:** Demographic characteristics of the study population

	Study population (*n* = 34)
Age, median (range)	57 (27–70)
Sex, male no. (%)	23 (67.6)
BMI (range)	26.0 (20.3–32.9)
Indoor stay time (hours)	24 (8–24)
Allergy Hx., no. (%)	9 (26.5)
Allergy FHx.	11 (32.4)
Atopy. no. (%)^a^	10 (32.3%)
Smoking, no. (%)	
Never	23 (67.6%)
Former	8 (23.5%)
Current	3 (8.8%)

^a^For the 31 patients who were available for atopy tests

**Table 2:** Ambient levels of volatile organic compounds (VOCs) in the old and new buildings

VOCs (μg/m^3^)		Old building	New building
TVOC		119	1054
5VOC	Benzene	0.86	41.94
	Toluene	13.5	235.15
	Ethylbenzene	0.36	1.54
	m, p Xylene	0.68	5.8
	Styrene	0.38	1.15
	o-Xylene	0.25	2.56
Formaldehyde		18.65	90.67
Acetaldehyde		3.8	12.99

**Fig. 2:**
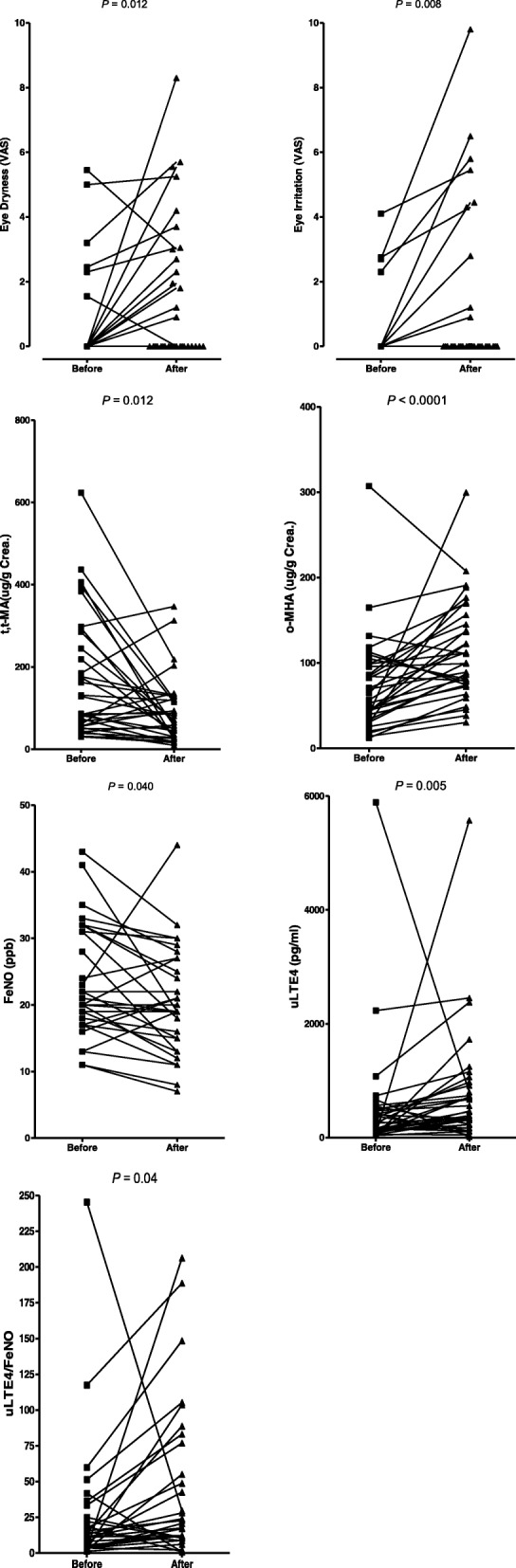
Changes in symptoms and urine levels of volatile organic compound (VOC) metabolites, leukotriene E4 (LTE4), and fractional exhaled nitric oxide (FeNO) before and after the move

The severity of eye dryness and eye irritation increased (*p* = 0.012 and *p* = 0.008, respectively), otherwise there were no changes in the severity of other symptoms. The urinary level of tt-MA decreased after the move (*p* = 0.012; Table 3; Fig. 2). In contrast, the levels of o-MHA, m-MHA, and p-MHA increased (*p* < 0.0001, *p* = 0.002, and *p* < 0.0001; Fig. 2). No changes were observed in the levels of the other VOC metabolites or oxidative stress markers before or after the move. No differences in the changes in the urinary markers were observed according to the eye symptoms after moving or based on a history of allergic diseases or the presence of atopy.

**Table 3:** Changes in lung function, inflammatory markers, and biochemical analyses

	Before move (*n* = 34)	After move (*n* = 34)	*p*-value
Lung function tests^a^			
FEV1, L	2.51 (0.69–4.05)	2.44 (1.39–3.96)	0.688
FEV1, %	90 (30–119)	93 (64–118)	0.410
FVC, L	3.14 (1.01–5.06)	3.03 (1.82–5.19)	0.688
FVC, %	90 (33–118)	91 (70–111)	0.448
FEV1/FVC	80 (65–89)	80 (67–90)	0.734
FEV25–75, L/s	2.71 (0.39–6.08)	2.57 (0.72–4.95)	0.899
FEV25–75, %	90 (19–168)	94 (33–190)	0.959
FeNO (ppm)	21 (11–43)	19 (7–44)	**0.040**
uLTE4 (pg/ml)	252.4 (36.8–5889.5)	460.6 (15.0–5570.9)	**0.005**
FeNO / uLTE4	11.1 (1.2–145.4)	20.7 (0.7–206.3)	**0.004**
Oxidative stress biomarkers			
MDA (μmol/g Cr.)	1.3 (0.4–8.8)	1.4 (0.4–3.3)	0.578
8-OHdG (ng/mg Cr.)	3.4 (0.6–8.2)	3.0 (0.3–8.3)	0.351
VOC metabolites			
t,t-MA (μg/g Cr)	85.8 (30.2–623.8)	68.0 (9.5–347.0)	**0.012**
HA (mg/g Cr)	204.4 (8.6–1984.9)	272.9 (18.0–1978.1)	0.714
MA (μg/g Cr)	183.5 (68.7–440.6)	215.1 (71.3–2481.6)	0.118
PGA (μg/g Cr)	47.4 (4.9–431.6)	63.4 (3.9–431.8)	0.966
o-MHA (μg/g Cr)	60.7 (12.2–307.0)	99.7 (30.3–299.6)	**0.000**
m-MHA (μg/g Cr)	101.7 (25.9–912.2)	164.6 (80.4–453.6)	**0.002**
p-MHA (μg/g Cr)	52.4 (17.5–401.3)	88.6 (30.2–630.9)	**0.000**

^a^Median (range)

† Sputum eosinophilia, eosinophil ≥3% in induced sputum

*AR* allergic rhinitis

Bold typeface indicates *P* < 0.05

FeNO decreased after the move (*p* = 0.04) and the levels of uLTE4 and uLTE4/FeNO increased (*p* = 0.005 and p = 0.04, respectively; Fig. 2). Although no changes in lung function were detected before and after the move (Table 3), the changes in FeNO were negatively correlated with the changes in forced expiratory volume in 1 s (FEV_1_) (L) and FEV_1_ (%) (*r* = − 0.376, *p* = 0.037 and *r* = − 0.505, *p* = 0.004, respectively; Additional file 1: Figure S1), and with the changes in forced vital capacity (FVC) (L) and FVC (%) before and after the move (*r* = − 0.387, *p* = 0.032 and *r* = − 0.407, *p* = 0.023, respectively; Additional file 1: Figure S1). However, no significant correlations were observed among the changes in urinary levels of VOC metabolites, oxidative stress markers, changes in the inflammation markers, including uLTE4, or the lung function tests.

## Discussion

This study showed changes in inflammatory markers before and after a move into a new building that had higher levels of ambient VOCs and formaldehyde. Decreased FeNO and an increase in uLTE4 after the move suggested changes in the inflammatory environment of the airway, possibly increased non-Th2 inflammation.

Some patients with asthma insist that their respiratory symptoms become aggravated after they move into a new house and point to the new indoor environment as a trigger of their symptoms. Actually, several studies have linked VOC exposure to asthma and other respiratory symptoms, although there is no consistent association between the total concentration of VOCs and SBS [1]. Nocturnal breathlessness in the general population is related to indoor concentrations of formaldehyde and VOCs [12]. VOC exposure and airway infections in infants are correlated [13]. A cross-sectional observation study reported the relationship among urinary levels of VOC metabolites, oxidative stress, and decreased lung function [5]. However, there is a lack of evidence for the direct effects of VOC exposure and changes in lung physiology in humans. This longitudinal study showed that 7-day exposure to ambient VOCs in daily life affected airway inflammation and possibly lung mechanics in the general population.

The FeNO measurement is used to assess the degree of Th2 allergic inflammation in the airway [14]. Decreased levels of FeNO after the move suggested a decreased level of Th2 inflammation in the airway and may suggest a relative increase in non-Th2 inflammation in the airway with increased uLTE4. Nitric oxide (NO) is present in all mammalian organs and is one of the endogenous regulators in physiological condition such as vasodilation and bronchodilation. NO increases during acute and chronic inflammation as an important inflammatory mediator [15–17]. NO in expired air is produced in bronchial epithelial cells by nitric oxide synthase (NOS)2, which increases in response to inflammation, and is thought to indirectly reflect airway inflammation [18]. Exhaled NO is associated with eosinophilic inflammation in the airway and is significantly correlated with eosinophil cationic protein and eosinophil counts in an airway mucosal biopsy and bronchial lavage of patients with asthma [19–21]. In the current study, the changes in FeNO were negatively correlated with the changes in FEV_1_ and FVC after the move, although no significant changes in lung functions were observed before or after the move. These results suggest that exposure to a higher dose of ambient VOCs during ordinary life inflamed the airway and induced changes in lung dynamics.

uLTE4 is a biomarker for assessing changes in the rate of total body cysteinyl leukotriene levels. Leukotrienes are a family of lipid mediators derived from arachidonic acid through the 5-lipoxygenase pathway that are produced by various leukocytes [10], and induce bronchoconstriction of the airway [22]. Approximately 5% of airway cysteinyl leukotrienes are eventually eliminated in the urine, and almost all in the form of uLTE4 [23]. Piedimonte et al. reported that uLTE4 was eight-fold higher in infants with bronchiolitis than in controls [24]. Among patients with asthma, uLTE4 is a biomarker of exposure to asthma triggers, such as air pollution and second-hand smoke, and is regarded as an important mediator of asthma exacerbation [10]. In addition, a high ratio of uLTE4 to FeNO (uLTE4/FeNO) seems to indicate predominantly non-Th2 airway inflammation [10]. Thus, increases in uLTE4 and uLTE4/FeNO in the current study suggest an increase in non-Th2 inflammation in the airway of the study population after moving into the new building.

Several studies have investigated human exposure to VOCs [25, 26]. These studies conducted short-term (2–4 h) exposure with 25–50 times higher (25–50 mg/m^3^) concentrations of VOCs and there were no changes in lung function or inflammatory markers, such as inflammatory cell profiles in nasal lavage and induced sputum, or biomarkers of airway inflammation, including LTB4 [26]. Several studies using a mouse model of asthma reported that exposure to a low dose of VOCs (several to 100 times the indoor air quality standard) increases allergic lung inflammation as adjuvants [27–30]. On the other hand, other animal studies have shown that exposure to VOCs is related to reactive oxygen species (ROS) and increased NOS [31, 32]. Subchronic exposure to low-dose VOCs in a mouse model induces the inflammatory response and release of ROS and mediators from activated eosinophils, neutrophils, alveolar macrophages, and epithelial cells [31]. Short-term exposure to four types of VOCs in a mixture (formaldehyde, benzene, toluene, and xylene) induces NOS activity and the NO signaling pathway in mice [32]. However, the current study showed that 7-day exposure to a low concentration of VOCs (1 mg/m^3^ total VOCs) in daily life decreased FeNO and increased uLTE4 in the normal population, suggesting increased non-Th2 inflammation in the airway. Differences between results of animal studies and the current study may have originated from differences in the concentration and duration of exposed VOCs and differences between mice and humans.

Yoon et al. showed that urinary levels of HA and MHA are associated with reduced lung function and that VOC metabolites are related with oxidative stress [5]. Toluene, o-, m-, and p-xylenes, styrene, and ethylbenzene are widely used as organic solvents in the plastic, rubber, and pharmaceutical industries. Major metabolites of these VOCs are HA, o-, m-, and p-MHAs, MA, and PGA, and their urinary concentrations have been used to biologically monitor occupational exposure to these VOCs [9]. The level of urinary MHA increased 7 days after moving into the new building. However, the level of urinary tt-MA decreased despite the move to a new indoor environment with higher ambient benzene. The reason for the decreased levels of urinary tt-MA is not clear but the absence of an association between the level of ambient benzene and urinary tt-MA was observed in a previous study [5]. About 2% of the total benzene taken up by humans is excreted via urine as tt-MA, and its excretion half-life is < 6 h [33]. Although urinary tt-MA has been recommended as a biomarker to assess occupational exposure to benzene, Jalai et al. showed that tt-MA cannot be used as a reliable biomarker for exposure to low dose benzene [34].

This study had several limitations that should be discussed. First, there were no direct evaluations of airway inflammation. Accessing airway inflammation is difficult, as most of the tools, including bronchoscopy and induced sputum, are invasive and require additional special support. Thus, several indirect methods used to evaluate airway inflammation have been tried. FeNO is a successful and commonly used method to access airway inflammation. uLTE4 is a laboratory tool used to access airway and non-Th2 inflammation (Gaki et al. 2007; Hallstrand and Henderson 2010; Kumlin et al. 1992; Pappas et al. 2000; Piedimonte et al. 2005; Rabinovitch 2012). Second, there could be many other variables that changed during the move into a new building, and the ambient levels of indoor VOCs constitute one of them. However, the outdoor environment was suspected to be similar before and after the move, as the distance between the new and old hospital was close, and the new hospital was located farther from the traffic. In addition, there were no large differences in temperature or air pollution around each evaluation date before and after the move. The move-in building was newly built, and both the old and new buildings had a similar structure as hospital buildings, with complete separation from secondary cooking products. In addition, personal factors, including age, smoking, medication, and weekly lifestyle, might be adjusted, as the same person was evaluated before and after the move at a similar time of day with a 14-day gap. Thus, changes before and after the move were suspected to be relatively limited to changes in the indoor environment, and exposure to higher levels of indoor VOCs were one of the main changes.

## Conclusions

Although VOCs in ordinary indoor environments are associated with respiratory symptoms, oxidative stress, and decreased lung function, no longitudinal study has shown the effects of VOC exposure to the airway in ordinary indoor environments. For the first time, this study showed that 7-day VOC exposure during ordinary life can affect airway inflammation, possibly non-Th2 inflammation.

## Additional file

Additional file 1 : **Table S1**. Outdoor environment over the 6 days before and at each evaluation date before and after the move. **Figure S1**. Correlation between changes in fractional exhaled nitric oxide (FeNO) and lung function tests before and after the move. (DOCX 62 kb)

## Abbreviations

8-OHdG: 8-oxo-29-deoxyguanosine
FeNO: Fractional exhaled nitric oxide
HA: Hippuric acid
MA: Mandelic acid
MDA: Malondialdehyde
o-, m- and p-MHAs: o-, m- and p-methylhippuric acids
PGA: Phenylglyoxylic acid
SBS: Sick building syndrome
Th2: T-helper cell type 2
tt-MA: tt-muconic acid
uLTE4: Urinary leukotriene E4
VAS: Visual analogue scale
VOC: Volatile organic compounds
