# Supplementary material for: Exposure to volatile organic compounds and airway inflammation
Source: Environ Health. 2018 Aug 7;17:65. doi: 10.1186/s12940-018-0410-1 (PMC6081941; doi:10.1186/s12940-018-0410-1)
Supplement: Supplementary file 1 — : Table S1. Outdoor environment over the 6 days before and at each evaluation date before and after the move. Figure S1. Correlation between changes in fractional exhaled nitric oxide (FeNO) and lung function tests before and after the move. (DOCX 62 kb) [file 12940_2018_410_MOESM1_ESM.docx]

Table S1. Outdoor environment over the 6 days before and at each evaluation date before and after the move

|  | ***Before move*** | ***After move*** | ***p-value*** |
| --- | --- | --- | --- |
| **Meteorological data** |  |  |  |
| Mean temperature (°C) | 11.7 (8.2–14.8) | 8.6 (3.2–13.9) | 0.128 |
| Max temperature (°C) | 17 (14.9–22.8) | 13.2 (10.2–19.2) | 0.011 |
| Mean humidity (%) | 68.9 (55.3–82.0) | 65.9 (52.3–97.8) | 0.535 |
| **Air pollution data** |  |  |  |
| PM_10_ (μg/m^3^) | 39 (23–57) | 38 (27–44) | 1.000 |
| O_3_ (ppm) | 0.018 (0.015–0.021) | 0.014 (0.009–0.027) | 0.383 |
| NO_2_ (ppm) | 0.016 (0.013–0.024) | 0.018 (0.014–0.028) | 0.805 |
| SO_2_ (ppm) | 0.003 (0.002–0.004) | 0.003 (0.002–0.004) | 1.000 |
| CO (ppm) | 0.6 (0.4–0.7) | 0.8 (0.7–1.1) | 0.001 |

Median (range)

Figure S1. Correlation between changes in fractional exhaled nitric oxide (FeNO) and lung function tests before and after the move
